# Measuring Adherence: A Proof of Concept Study for Multiple Medications for Chronic Conditions in Alternative Payment Models

**DOI:** 10.3390/pharmacy7030081

**Published:** 2019-07-02

**Authors:** Joel F. Farley, Arun Kumar, Benjamin Y. Urick

**Affiliations:** 1 Department of Pharmaceutical Care and Health Systems, College of Pharmacy, University of Minnesota, Minneapolis, MN 55455, USA; 2 Center for Medication Optimization, UNC Eshelman School of Pharmacy, University of North Carolina, Chapel Hill, NC 27599, USA

**Keywords:** medication adherence, quality measurement/benchmarking, multiple chronic conditions, CMS Star rating

## Abstract

Adherence to renin angiotensin system antagonists (RASA), non-insulin diabetes medications (NIDM) and statins has been included in the Medicare Star Ratings program since 2012. The long-term use of these measures emphasizes adherence to a limited number of chronic medications and may present opportunities for Part D plan sponsors to misuse the measures to influence their Medicare Part D Star Rating. It also does not capture the adherence needs of high-risk patients with multiple chronic conditions. The objective of this study was to describe the development of a new measure to capture adherence to multiple medications for chronic conditions (MMCC). The MMCC measure captures adherence to 71 different therapeutic categories of medication and was constructed using North Carolina Medicaid prescription claims data from 2015 to 2017. This measure was validated against the existing RASA, NIDM and statin adherence measures. This new measure was highly correlated with Star Rating measures, captured a greater number of eligible patients than these existing measures and had a lower proportion of patients meet the adherence threshold than the existing Star Ratings adherence measures. There is an opportunity to develop new measures, which include adherence to multiple medications in populations with multiple chronic conditions.

## 1. Introduction

### 1.1. Medication Adherence and Multiple Chronic Conditions

Multiple chronic conditions (MCC), defined as the presence of two or more concurrent chronic conditions, presents a significant burden to the U.S. health care system. It is estimated that 50% of older adults have three or more concurrent chronic medical conditions [1]. The burden of MCC is felt by the health care system through increased health service utilization and expenditures. It is estimated that 66% of total U.S. health care spending is directly attributable to a disproportionally small number of approximately 27% of American’s with MCC [2]. This burden is felt not only by the health care system, but also by patients through higher morbidity and mortality as well as reductions in patient reported quality of life [3] (p. 15).

The primary treatment modality for most chronic conditions comes in the form of prescription medication treatments. It is estimated that 60% of elderly patients are prescribed with three or more medications [4]. In reports from the U.S. Centers for Disease Control and Prevention (CDC), some 37% of older Americans are reported to use five or more prescription medications concurrently with this prevalence doubling since 1999 [5]. As the number of medications used by a patient to manage chronic conditions increases, so too does the risk of inappropriate medication use and adverse drug events [6,7].

Ensuring medications are used appropriately is imperative to reducing the burden of MCC. However, estimates of non-adherence to medications are reported to be as high as 50% for many chronic conditions [8,9]. Medication non-adherence is responsible for an estimated 33–69% of medication-related hospitalizations [10] and may result in an additional $300 billion in direct health care spending in the U.S annually [11]. Further evidence provided by the CDC suggests medication non-adherence is tied to approximately 50% of treatment failures in chronically ill patients and corresponds to approximately 125,000 deaths per year [12].

### 1.2. Adherence and Star Ratings

Given the importance of medication adherence to the quality of care provided to patients, a number of initiatives have been taken to improve rates of adherence to medications for chronic medical conditions. One initiative adopted by the Centers for Medicaid and Medicare Services (CMS) is to tie health plan payments and performance ratings to their population’s medication adherence rates for a select number of chronic health conditions using the CMS Star Ratings program. Beginning in 2007, the Star Ratings program was implemented as a means for CMS and Medicare beneficiaries to assess and compare plans on their performance with a broad array of measures associated with the quality of care provided to patients. For standalone prescription drug plans (PDPs), these performance metrics provide a benchmark for patients to select health plans on the basis of the quality of care provided. For Medicare Advantage plans offering health and prescription drug benefits, these performance metrics have additional importance and have been tied to per member per month (PMPM) quality bonus payment rates since 2012 [13].

The metrics used to measure plan performance change from year to year with the 2018 Medicare Star Ratings including 48 different quality and performance measures, of which 14 involve ratings of prescription drug plan indicators [14]. Three of the 14 prescription drug plan indicators in the Star Ratings program measure medication adherence using a prescription claims-based measure of adherence termed the proportion of days covered (PDC). Under the Star Ratings program, patients are considered adherent to treatment if they have a PDC of 80% or higher and fill two or more prescriptions in a year for any of the following three chronic medication therapeutic categories: Renin angiotensin receptor antagonists (RASA) for hypertension, non-insulin diabetes medications (NIDM) and/or statin medications for hyperlipidemia. Each of these three medication adherence ratings is triple weighted in the Star Ratings program demonstrating their importance to achieving a summary Star Rating for the health plan, which can range anywhere from one to five Stars per plan year [15].

Although different metrics under the Star Ratings program churn over time, the three medication adherence measures used to evaluate plan performance have been in place in one form or another since 2012 [16]. The consistency of these measures in the Star Ratings program, as well as the heavy weights placed on these outcomes, leads to a heavy emphasis by health plans in achieving adherence targets. As a result, the trend in adherence to these medication therapeutic categories has increased significantly over time. From 2017 to 2019, an MAPD plan receiving five Star status for NIDM, RASA and statin medications saw an increase from 83% to 85%, 83% to 88% and 82% to 87% respectively [17]. Over the same time-period, a four star MAPD plan’s targets increased for these same medication categories from 79% to 81%, 79% to 86% and 77% to 83% respectively.

### 1.3. Limitations of Star Ratings Adherence Measures

Improvements in medication adherence rates reported through the Medicare Star Ratings program are encouraging, but also raise a number of important questions. The trend in rates of adherence now exceeds what is commonly accepted as the true rate of medication adherence in the clinical literature. A recent study of more than 200,000 diabetic patients showed that only 69% or oral diabetes medication users are 80% or more adherent to their medication when measured using the medication possession ratio (MPR), which is a more liberal measure of adherence than the Star Ratings’ PDC. [18]; another recent study, which including 238,372 patients using different oral anti-diabetes medications, suggested that 47.3%, 41.2% and 36.7% of patients were adherent to DPP4 inhibitors, sulfonylurea medications and thiazolidinedione therapies respectively when measured as a PDC of 80% or higher during a one year follow up period [19]. Similar findings are present when looking at other medication classes. Even in a large population of high-risk Medicare beneficiaries with a previous myocardial infarction (MI), the rate of adherence to statin medications as measured by a PDC of 80% or higher one year after their MI was only 59% at six months and 42% at two years in patients 66 to 75 years of age [20]. Combined, our review of the clinical literature suggest that the true rate of medication adherence to therapeutic classes included in the Star Ratings program is lower than that reported by CMS.

The discrepancy in Star Ratings-reported adherence rates and the rates commonly found in the clinical literature raise a number of important questions. The first question relates to the validity of the PDC as a measure of adherence when applied to performance-based value incentive models for payment. It is important to remember that the PDC measure does not measure actual consumption of medication by patients, but instead measures patterns of refills using prescription claims data. In essence, gaps between an actual fill date and an expected fill date, which is based on days supply from a prior fill, are counted as “uncovered” days in the PDC measurement. These gaps in coverage are compared to the length of time a person is observed to account for the proportion of days covered on treatment. Although the PDC has been validated against other commonly used measures of medication adherence such as patient self-report and medication electronic monitoring systems (MEMS) [21,22], they do not measure actual consumption of medication by patients and are imperfect metrics of actual adherence.

In addition, claims based adherence metrics may be subject to gaming by health plans attempting to improve Star Ratings and quality bonus payments. The use of automatic refill programs is one example of an early strategy employed by health plans to potentially gain higher PDC rates. Sending a refill to patients without their need to authorize the next fill reduces the risk that a patient will not fill their next medication from the pharmacy, but does not actually ensure in any way that once a patient receives their medication they will continue to use it. To reduce waste associated with shipping unneeded medication to patients, CMS implemented policies limiting this practice in 2013 [23,24] (p. 133).

The use of mail order programs and 90-day fills are other potential strategies to improve PDC rates even if not directly associated with improvements in patient medication consumption. Since the PDC relies on the need to identify gaps between fills to identify periods of non-adherence, reducing the number of potential gaps over a measurement period may artificially inflate PDC rates [25]. Over a 12-month period, the number of potential gaps is three with 90-day fills compared to eleven with 30-day fills. The use of 90 day fills, which are common in mail order programs, may result in artificial improvements in adherence that are not reflective of actual improvements in medication consumption patterns by patients.

Even if one assumes the PDC rates reported in the Star Ratings program reflects a valid rate of medication adherence in patients, the very high rates of adherence reported by health plans still raise concerns. First, the consistent use of the same measures over time means that health plans adopting programs to improve medication use in Medicare are focusing clinical efforts on a limited number of chronic conditions. As previously mentioned, it is estimated that 50% of older adults have three or more concurrent chronic conditions at any given time [1]. Focusing on a select number of these conditions does not address the entire scope of medical conditions a patient may have and may resulted in siloed clinical offerings by health plans in an attempt to improve plan ratings. In addition, the rates of adherence in four and five Star Plans are approaching the ceiling of the PDC measurement, which caps at 100%. This reduces the ability to differentiate high performing plans from low performing plans.

Given the potential limitations of the current medication adherence measures in the CMS Star Ratings program, opportunities to develop and validate new measures of medication adherence may be useful. The objective of this study is to describe the development of a measure of medication adherence for patients using multiple medications for chronic conditions. This measure was developed to incentivize payment to community pharmacies to improve medication adherence for a broad range of chronic conditions through the development and delivery of enhanced pharmacy services [26,27].

## 2. Materials and Methods

### 2.1. Setting

The development of a new measure designed to capture adherence to multiple medications for chronic conditions (MMCC) was used to support payment incentives under an alternative payment model (APM) in a network of community pharmacies providing enhanced services to Medicaid recipients in North Carolina. This program was supported by a CMS Innovations Award provided to Community Care of North Carolina, which serves as the primary care-based medical home for the state’s Medicaid population. The specific aspects of this APM are described elsewhere [28]. In brief, the APM model included four different medication adherence metrics (RASA, NIDM, statin and MMCC) as well as three health service utilization measures (emergency department utilization, inpatient hospital visits and total cost of care) to measure the performance of each pharmacy relative to their peers. The MMCC measure described herein is one of the four adherence measures used to incentivize pharmacy performance.

### 2.2. Data

We constructed our measure of MMCC using North Carolina Medicaid prescription claims data from 2015 through 2017. As part of the APM, the measure was constructed on a quarterly basis with a rolling 12-month look back period to facilitate payment. Additional Medicaid claims data for outpatient and inpatient services as well as beneficiary enrollment data were also used to attribute patients to the network and facilitate payment. This study was not considered as human subjects research and was exempted from Institutional Review Board approval.

### 2.3. Sample

To be considered for inclusion into the MMCC measure, patients must first meet attribution criteria to the APM. Eligibility criteria included continuous Medicaid program enrollment for the entire calendar year (2015, 2016 or 2017). We further required that patients not be dually enrolled into the Medicare program to ensure we were able to fully capture all prescription drug claims. Finally, we limited the analysis to patients 18–64 years of age.

### 2.4. Medication Adherence Measurement

We used a list of 71 different therapeutic categories constructed by CCNC to identify therapeutic categories for inclusion in the MMCC measure. These categories were constructed by CCNC to identify high-risk chronic conditions effecting the Medicaid population. As seen in Table 1, the chronic conditions included in our measure spanned a wide variety of different therapeutic areas including chronic infectious conditions (e.g., hepatitis and HIV), diabetes, hypertension, additional cardiovascular conditions (e.g., diuretics, angina, anticoagulation), mental health conditions (depression, schizophrenia, bipolar disorder), osteoporosis, seizures and a number of other chronic conditions. This measure is much more inclusive of overall types of conditions patients might present with than the three Star Ratings adherence measures currently in use. Given that this list was constructed for purposes other than simply the construction of the MMCC adherence measure and applies specifically to a Medicaid population, which may require care management services, this study represents primarily a proof-of-concept exercise.

We measured adherence using PDC. A specific PDC was calculated for each of the 71 therapeutic categories of medications and followed the Star Ratings technical specifications. Hospitalized days were accounted for by crediting the patient for a medication believed to be given in the hospital if the patient was covered by the medication on the day they were admitted [29]. To be eligible for the MMCC measure, patients were required to use four or more of the 71 chronic therapeutic categories of medication from Table 1 over the 12 month calendar year period. In addition to the MMCC measure, we also constructed annual medication adherence rates for the three Star Ratings adherence measures (RASA, NIDM and statins) to evaluate correlations between the new measure and these established metrics. Patients were deemed adherent to the Star Ratings measures if their PDC was 80% or higher. To be considered adherent to the MMCC measure, we required patients to have a PDC of 80% or higher for at least 75% of the therapeutic categories used. For example, a patient using four specific therapeutic categories would be deemed adherent if they had a PDC of 80% or higher for at least three of the four therapeutic categories.

### 2.5. Criterion Validity Testing

We examined the potential criterion validity of the newly developed MMCC measure by correlating the measure with the three existing Star Ratings measures, which have gone through prior validation. This validity testing process assumes that adherence to Star Rating adherence measures and the MMCC measure are positively correlated. Given that the MMCC measure is constructed within each year (2015, 2016 and 2017) we first correlated the MMCC measure with each of the three Star Ratings measures using chi-square testing within the year of analysis. The chi-square comparison examines whether or not patients who were adherent to the MMCC measure (>75% of chronic medications used had a PDC ≥ 80%) were also categorized as adherent to the Star Ratings measure of interest (PDC ≥ 80%) Next, we assessed the correlation between MMCC and Star Ratings measures by running generalized estimating equations (GEE) models, which controlled for repeated observations for patients contributing adherence information across more than one single year of data to control for within subject variation. The GEE model specifies a binomial distribution with a log link. Data are converted to odds-ratios and are presented with corresponding 95% confidence intervals. All statistical analyses were deemed significant at α = 0.05 and the analyses were run in SAS Version 9.4 (Cary, North Carolina, USA).

## 3. Results

Across the three-year period, more than 40,000 patients per year were eligible for the MMCC measure (Table 2). The number of patients eligible for the MMCC measure exceeded the approximate 10,000 patients a year eligible for the NIDM measure and more than 20,000 patients eligible per year for RASA and statin measures. The mean number of MMCC-eligible therapeutic categories was six with a range from four to 22. In 2017, the percent of patients eligible for adherence measures for NIDM, RASA and Statin medications that were adherent to treatment as defined by a PDC ≥ 80% (51.3%, 44.0% and 51.2%) was higher than the percent of patients adherent to the MMCC measure (34.6%). This trend was consistent across all years.

Although unique from existing adherence measures, the three categories of medication that deem a patient eligible for inclusion into the Star Ratings program (RASA, NIDM and statins) were also common in patients eligible for the MMCC measure. Within each year, more than 66% of patients eligible for the MMCC measure were also eligible for inclusion into one or more of the Star Ratings adherence measurements. In 2017, 19.4%, 43.7% and 45.2% of patients eligible for MMCC measurement were also eligible for measurement of NIDM RASA, and statin medication adherence measures in the Star Ratings program respectively.

We hypothesized that among patients attributed to the MMCC measure who were also eligible for any of the three Star Ratings measures, adherence to the previously validated Star Ratings adherence measure would correlate with adherence to the new MMCC measure. Table 3 presents bivariate chi-square comparisons between each Star Ratings adherence measure and the MMCC measure. Overall, this correlation shows that among patients adherent to MMCC there is a higher likelihood of adherence to the three Star Ratings measures and similarly, among non-adherent MMCC patients there is a higher likelihood of non-adherence to Star Ratings measures. If a patient is not adherent to a Star Ratings PDC measure, they are unlikely to be adherent to the MMCC measure. For example, in 2017, only 6.9% of non-adherent NIDA users, 7.5% of non-adherent RASA users and 6.2% of non-adherent statin users were considered adherent for the MMCC measure. However, there were a significant number of patients deemed adherent to the Star ratings measures, which were not deemed adherent to the MMCC measure for each of the Star Ratings measures across each year. To illustrate, 50.7% of adherent NIDA users, 48.8% of adherent RASA users and 44.5% of adherent statin users were deemed not adherent to MMCC respectively in 2017.

Accounting for multiple observations across the three years, we further exaimine the relationship between adherence to the three existing Star Ratings measures and the probability of adherence to MMCC using GEE. Table 3 describes the odds-ratios and corresponding confidence intervals resulting from those tests. The odds ratio associated with MMCC adherence in adherent NIDA (Odds Ratio (OR) = 13.64: 95% CI: 12.6, 14.8), RASA (OR = 16.69: 95% CI: 15.8, 17.6) and Statin (OR = 21.32: 95% CI: 20.2, 22.6) users suggests a very strong correlation between the measures.

## 4. Discussion

Given the limitations associated with the three existing Star Ratings measures, we explored the implementation of a new MMCC measure of medication adherence. This measure captures more of the overall adherence needs of a patient by considering adherence to 71 different therapeutic categories of medication. This new measure has less opportunity for potential gaming by health plans, which are currently only required to emphasize adherence for a limited number of health conditions. As a more patient centered measure of medication adherence, the MMCC measure emphasizes more than a single chronic condition and is more reflective of the overall health needs of a patient. In addition, the focus on patients with multiple chronic conditions with the MMCC targets a high-risk population of patients with MCC who have higher than average health expenditures. Emphasizing adherence in this population is likely to pay greater dividends to actual health improvement than emphasizing adherence in patients with a single condition, some of which may be otherwise healthy.

The MMCC measures included more patients than any of the Star Ratings measures individually, and more than the total number across all Star Ratings measures combined. Additionally, there were many patients who were included in the traditional PDC measures who were not included in the MMCC measure. This suggests that the MMCC measure may target a different population than the traditional measures, and, because of larger denominators, be a more statistically reliable measure of medication adherence. Furthermore, the proportion of patients who met the adherence threshold for MMCC measure was substantially lower than the proportion of patients categorized as adherent to the traditional adherence measure. If confirmed in a Medicare population, this measure would solve some of the ceiling problems, which have been observed for four and five star plans.

To examine the criterion validity of this measure to previously validated adherence measurements, we correlated the MMCC measure with these three Star Ratings measures and found a very high correlation between them. However, we also found evidence that the criteria, which defined adherence to this new MMCC measure was more difficult to meet than the current criteria for NIDA, RASA or statin medication adherence. This suggests that the new measure may have less ceiling effect and provide better discriminative ability.

The results of this study should be interpreted in light of a number of important limitations. In many ways, this study represents a proof of concept investigation into the possibility of using an MMCC measure to capture medication adherence more broadly than that observed with limited therapeutic categories for single conditions. The therapeutic categories included in the measure were selected to support the needs of the state Medicaid program. This led to the inclusion of therapeutic categories that represent high-risk populations (e.g., HIV and Hepatitis) that might not represent the needs of commercially insured or Medicare plans to the same extent. For this measure to be accepted more broadly as a measure of plan performance, it is important to obtain the perspective of clinicians, patients and plans in the process of identifying which drug classes to include in an eventual MMCC measure. It should also undergo a formal validation process required of any new measure prior to implementation [30,31]. Finally, the proof of criterion validity in this study included the three Star Ratings measures, which we identified as having significant limitations in this article.

## 5. Conclusions

Given existing concerns associated with the use of medication adherence measures for limited therapeutic categories as part of the Star Ratings program in Medicare, opportunities for the development of new adherence performance measures exist. This proof of concept study shows the potential benefits associated with the development of a performance metric designed to capture adherence in patients with multiple chronic conditions. This measure correlated well with other validated adherence measures. Opportunities to develop and implement new quality metrics of medication adherence such as the MMCC should be explored.

## Figures and Tables

**Table 1:** Therapeutic categories included in initial multiple medications for chronic conditions (MMCC) adherence measurement.

Indication	Therapeutic Categories
Asthma	Anti-IgE Monoclonal Antibodies, Inhaled Corticosteroids, Leukotriene Modulators
Diabetes	Alpha-Glucosidase Inhibitors, Biguanides, Dipeptidyl Peptidase-4 (DPP-4) Inhibitors, Sulfonylureas, Thiazolidinediones
Hepatitis B	Hepatitis B agents
Hepatitis C	Inceivek, Interferon, Simeprevir, Ribavarin, Sofosbuvir, Boceprevir
HIV	Antiretrovirals (Entry inhibitors, integrase inhibitors, protease inhibitors, ritonavir) Antiretroviral Reverse Transcriptase Inhibitors (RTI) Non-nucleoside analogues, Antiretroviral RTI Nucleoside analogues (purine, pyrimidines, thymidines) Antiretroviral Nucleotide Analogues, Antiretroviral CMV agents, Cobicistat, Antiretroviral combination products
Hyperlipidemia	Antihyperlipidemics, Antihyperlipidemics-Bile Acid Sequestrants
Cardiovascular conditions	Antiadrenergic agents, Antianginal agents (non-nitrates), Antiarrhythmics, Antiplatelets
Hypertension	ACE Inhibitor, Alpha-beta blockers, Angiotensin II receptor antagonists, Beta-blockers, Calcium channel blockers, Renin inhibitors, Selective aldosterone receptor antagonists
Diuresis	Thiazide diuretics, Potassium sparing diuretics, Loop diuretics
ADHD	ADHD stimulants, ADHD miscellaneous
Dementia	Acetylcholinesterase inhibitors, NMDA receptor antagonists
Antidepressants	Alpha-2 Receptor Antagonists, Antidepressants-Combo, Antidepressants-Modified Cyclics, Serotonin-Norepinephrine Reuptake Inhibitors (SNRIs), Monoamine Oxidase Inhibitors (MAOIs), Selective Serotonin Reuptake Inhibitors (SSRIs)
Other Mental Health	Antipsychotics-Second Generation, Antipsychotic-First Generation, Antimanic Agents, Antiparkinson Agents
Seizures	Anticonvulsants misc.
Osteoporosis	Bone density Regulators-Bisphosphonates and Calcitonins, Bone density Regulators-Parathyroid Hormone, Vitamin D, Selective Estrogen Receptor Modulators
Contraception	Contraceptives misc.
Digestive Aids	Digestive enzymes misc.
Gout	Gout agents misc.
Thyroid Disorder	Hyperparathyroid Treatment-Vitamin D Analogs, Hypothyroid agents
Prostatic hypertrophy	Prostatic Hypertrophy Agents
Peptic ulcer	Peptic ulcer agents misc.

**Table 2:** Descriptive statistics of adherence measurement for full sample and MMCC ^1^ eligible patients.

	2015	2016	2017
**Full Sample Eligible Population**	N = 93,857	N = 96,824	N = 83,711
Eligible for NIDM ^2^ Adherence Measure	9681 (10.3%)	10,669 (11.0%)	9394 (11.2%)
Eligible for RASA ^3^ Adherence Measure	27,495 (29.3%)	28,853 (29.8%)	23,244 (27.8%)
Eligible for Statin Adherence Measure	23,201 (24.7%)	25,044 (25.9%)	21,565 (25.8%)
Adherent (≥ 80% PDC) NIDM Users	5101 (52.7%)	5988 (56.0%)	4821 (51.3%)
Adherent (≥ 80% PDC) RASA Users	15,030 (54.7%)	15,844 (54.9%)	10,242 (44.0%)
Adherent (≥ 80% PDC) Statin Users	12,996 (56.0%)	13,573 (54.2%)	11,030 (51.2%)
**MMCC ^1^ Eligible Population**	N = 43,712	N = 46,813	N = 40,001
Therapeutic Categories Defining Eligibility			
4 therapeutic categories	12,021 (27.5%)	12,520 (26.7%)	11,197 (28.0%)
5 therapeutic categories	9564 (21.9%)	10,194 (21.8%)	8899 (22.3%)
6 therapeutic categories	7192 (16.5%)	7747 (16.6%)	6680 (16/7%)
7 therapeutic categories	5353 (12.3%)	5849 (12.5%)	4787 (12.0%)
8 or more therapeutic categories	9582 (21.9%)	10,503 (22.4%)	8438 (21.1%)
Mean Count of Therapeutic Categories (SD)	6.1 (2.1)	6.1 (2.2)	6.1 (2.1)
Range of Therapeutic Categories	(4,21)	(4,22)	(4,21)
Also Eligible for Star Rating Adherence Measure	29,032 (66.4%)	31,343 (67.0%)	26,487 (66.2%)
Also Eligible for NIDM ^2^ Adherence Measure	8068 (18.5%)	8912 (19.0%)	7754 (19.4%)
Also Eligible for RASA ^3^ Adherence Measure	20,371 (46.6%)	21,651 (46.3%)	17,464 (43.7%)
Also Eligible for Statin Adherence Measure	19,460 (44.5%)	21,154 (45.2%)	18,063 (45.2%)
Adherent MMCC Users ^4^	15,114 (34.6%)	15,133 (32.3%)	11,679 (29.2%)
MMCC Users Adherent to NIDM ^2^ (≥ 80% PDC)	4525 (56.1%)	5287 (59.3%)	4229 (54.9%)
MMCC Users Adherent to RASA ^3^ (≥ 80% PDC)	11,679 (57.3%)	12,387 (57.2%)	7998 (46.0%)
MMCC Users Adherent to Statin (≥ 80% PDC)	11,190 (57.5%)	11,722 (55.4%)	9515 (52.7%)

^1^ MMCC = Multiple Medications for Chronic Conditions; ^2^ NIDM = Non-Insulin Diabetes Medications; ^3^ RASA = Renin Angiotensin System Antagonists; ^4^ MMCC Adherence Rate is defined as ≥ 75% of medications with ≥ 80% PDC.

**Table 3:** Correlational testing between MMCC and existing Star Ratings adherence measurements.

		Chi-square Correlational Testing Between Star Ratings and MMCC Adherence Measures									Generalized Estimating Equation Testing Across All Years	
		2015		*p*-value	2016		*p*-value	2017		*p*-value		
		MMCC ^1^ Adherence			MMCC Adherence			MMCC Adherence				
		< 75%	≥ 75%		< 75%	≥ 75%		< 75%	≥ 75%		Odds Ratio	*p*-value
											(95% CI)	
NIDA ^2^ Adherence	< 80%	3198 (39.6%)	345 (4.28%)	< 0.001	3363 (37.7%)	262 (2.9%)	< 0.001	3237 (42.2%)	238 (3.1%)	< 0.001	13.64 (12.6, 14.8)	< 0.001
	≥ 80%	1912 (23.7%)	2613 (32.4%)		2394 (26.9%)	2893 (32.4%)		2146 (27.9%)	2083 (27.0%)			
RASA ^3^ Adherence	< 80%	8199 (40.3%)	493 (2.4%)	< 0.001	8746 (40.4%)	518 (2.4%)	< 0.001	8670 (49.9%)	706 (4.1%)	< 0.001	16.69 (15.8, 17.6)	< 0.001
	≥ 80%	5281 (25.9%)	6398 (31.4%)		5947 (27.5%)	6440 (29.7%)		3905 (22.5%)	4093 (23.6%)			
Statins Adherence	< 80%	7775 (40.0%)	495 (2.5%)	< 0.001	8763 (41.4%)	669 (3.2%)	< 0.001	8016 (44.4%)	532 (3.0%)	< 0.001	21.32 (20.2, 22.6)	< 0.001
	≥ 80%	4140 (21.3%)	7050 (36.2%)		4736 (22.4%)	6986 (33.0%)		4238 (23.5%)	5277 (29.2%)			

^1^ MMCC = Multiple Medications for Chronic Conditions;^2^ NIDA = Non-Insulin Diabetes Adherence Measure;^3^ RASA = Renin Angiotensin System Antagonists.
